# Critical Issues in the Assessment of Loneliness in Older Latino Adults in the Dolores and Soledad Qualitative Study

**DOI:** 10.1093/geroni/igaf122.060

**Published:** 2025-12-31

**Authors:** David Camacho, Kelly Pacheco, María Aranda, M Carrington Reid, Elaine Wethington

**Affiliations:** University of Illinois Chicago, Chicago, Illinois, United States; New York University, New York, New York, United States; University of Southern California, Los Angeles, California, United States; Weill Cornell Medicine, New York, New York, United States; Cornell University, Ithaca, New York, United States

## Abstract

Despite the large number of US older Latinos, there is a dearth of literature that examines loneliness in this population. We examine loneliness assessment issues from the Dolores and Soledad Study that included loneliness and/or chronic pain illness narrative interviews with 35 Spanish-speaking Latinos (60 years+) with multiple chronic conditions. Loneliness assessment included the question “Do you feel lonely?” and the 3-item UCLA Loneliness scale. Interviewers were bilingual (English/Spanish) gerontological social workers with training in culturally sensitive clinical research, and elicitation of “sensitive data.” Participants were predominantly Puerto Rican or immigrated from Dominican Republic, Ecuador, and Mexico. Most participants had lived in the US (New York or Los Angeles) for 30+ years, and overwhelmingly reported clinically significant depressive symptoms or chronic pain (86%). We identified multiple assessment challenges including participants’: 1) hesitation about discussing emotional well-being; 2) limited prior discussion of loneliness with family, friends or professionals (e.g., to prevent their emotional distress); 3) linguistic and conceptual difficulties in differentiating between loneliness, social isolation, and solitude that do not align with westernized perspectives (e.g., “soledad” may indicate loneliness and/or solitude and influenced by familism and immigrant experiences); 4) ambivalence about feelings of loneliness (e.g., felt they should not feel alone); 5) non-linear descriptions that included varying dimensions (e.g., emotional /existential, chronicity, causes); and 6) loneliness narratives that were linked to traumatic life events (e.g., physical/sexual violence). Findings support a need for culturally-informed loneliness assessments and narrative methods. These approaches may enhance trust and facilitate recognition and elicitation of loneliness narratives.

